# Socioeconomic and medical determinants of state‐level subjective cognitive decline in the United States

**DOI:** 10.1002/alz.14220

**Published:** 2024-10-01

**Authors:** Adam de Havenon, Eric L. Stulberg, Lauren Littig, Ka‐Ho Wong, Daniel Sarpong, Vivian Li, Richa Sharma, Guido J. Falcone, Jeff D. Williamson, Nicholas M. Pajewski, Rebecca F. Gottesman, Adam M. Brickman, Kevin N. Sheth

**Affiliations:** ^1^ Department of Neurology Center for Brain and Mind Health Yale University School of Medicine New Haven Connecticut USA; ^2^ Department of Neurology University of Utah Salt Lake City Utah USA; ^3^ Department of Population Health Science University of Utah Salt Lake City Utah USA; ^4^ Department of General Internal Medicine Center for Brain and Mind Health Yale University School of Medicine New Haven Connecticut USA; ^5^ Department of Internal Medicine Geriatrics and Gerontology and the Sticht Center for Healthy Aging and Alzheimer's Prevention Wake Forest University School of Medicine Winston‐Salem North Carolina USA; ^6^ Department of Biostatistics and Data Science Wake Forest University School of Medicine Winston‐Salem North Carolina USA; ^7^ National Institute of Neurological Disorders and Stroke Bethesda Maryland USA; ^8^ Department of Neurology Taub Institute for Research on Alzheimer's Disease and the Aging Brain Columbia University New York New York USA

**Keywords:** brain health, cerebrovascular pathology, cognitive impairment risk, subjective cognitive decline

## Abstract

**INTRODUCTION:**

It is important to understand the socioeconomic and medical determinants of subjective cognitive decline (SCD) at a population level in the United States.

**METHODS:**

The primary outcomes are state‐level rates of SCD and SCD‐related functional impairment in adults aged ≥ 45, both measured in the Behavioral Risk Factor Surveillance System from 2016 to 2022. The exposures are state‐level rates of poverty, unemployment, homelessness, college education, racial and ethnic minorities, uninsurance, smoking, hypertension, diabetes, and obesity as well as household income and physician density.

**RESULTS:**

The strongest state‐level associations with rates of SCD were the prevalence of diabetes (rho = 0.64), hypertension (rho = 0.59), and poverty (rho = 0.58; all *p* < 0.001), and with SCD‐related functional impairment were prevalence of poverty (rho = 0.71), diabetes (rho = 0.68), and hypertension (rho = 0.53; all *p* < 0.001).

**DISCUSSION:**

This study highlights critical links between SCD and socioeconomic and medical determinants in adults aged ≥ 45 in the United States, including the prevalence of poverty, diabetes, and hypertension.

**Highlights:**

- State‐level analysis reveals socioeconomic and medical risk factors for subjective cognitive decline (SCD) at a population level.
- The prevalence of poverty is a critical contributor to the state‐level prevalence of SCD.
- The prevalence of diabetes and hypertension are also strong state‐level determinants of SCD.
- Addressing the burden of cognitive decline at the population level necessitates targeting socioeconomic and medical factors.

## 1 BACKGROUND

The prevalence of dementia continues to rise.^1^, ^2^, ^3^, ^4^ Apart from the effect of population age on the societal risk of cognitive decline, prior research focused on individual risk factors such as the amyloid and tau pathology seen in Alzheimer's disease (AD), vascular risk factors including hypertension and diabetes, the impact of diet and exercise, and the protective effects of education.^5^, ^6^, ^7^, ^8^, ^9^, ^10^, ^11^, ^12^, ^13^ An emerging body of research identifies distinct socioeconomic and economic determinants that can impact an individual's likelihood of developing cognitive decline or dementia.^8^, ^9^, ^12^, ^13^, ^14^, ^15^, ^16^, ^17^, ^18^, ^19^ However, this research was conducted in cohorts, in part due to the limitations of existing datasets. Investigating regional factors may be important in informing localized public health initiatives aimed at enhancing brain health within communities.

A recent publication used models derived from a local research cohort and census statistics to produce estimates of dementia prevalence at the state and county level in the United States that were extrapolated from the National Center of Health Statistics and the Chicago Health and Aging Project.^20^ However, it lacked a direct measurement for dementia outside of their local cohort. The Centers for Disease Control and Prevention (CDC) measures self‐reported subjective cognitive decline (SCD) and SCD‐related functional impairment at the state level.^21^ While neither represent a diagnosis of cognitive impairment or dementia based on formal testing, they are predictive of a future diagnosis of cognitive impairment and can be more easily measured in populations.^22^, ^23^, ^24^, ^25^, ^26^, ^27^, ^28^, ^29^, ^30^ Our study aims to clarify the association between state‐level socioeconomic and medical determinants and state‐level prevalence estimates of SCD and SCD‐related functional impairment. We acknowledge the complex directionality and causal pathways that may co‐exist between state‐level measurements of socioeconomic characteristics and medical determinants (e.g., socioeconomic → SCD; socioeconomic → medical → SCD; medical → socioeconomic → SCD). Nonetheless, we believe this hypothesis‐generating approach is appropriate because it shifts the focus from individual risk factors in local cohorts to broader geopolitical characteristics that may be more amenable to public health intervention.

## 2 METHODS

### 2.1 Datasets

This is an ecological study that uses data from the time period 2016 to 2022 in the United States. The outcome variables are obtained from the CDC dataset “Alzheimer's Disease and Healthy Aging Data,” which contains state‐level Behavioral Risk Factor Surveillance System (BRFSS) surveys from 2016 to 2021.^31^ There is no information on individual subjects in this publicly released dataset. The BRFSS data used in this analysis were derived from the Cognitive Decline Module, which is a CDC‐developed tool for understanding SCD in adults and part of the larger National Brain Health Initiative.^32^ BRFSS uses a telephone survey to sample non‐institutionalized US residents randomly and gather information regarding their health‐related behaviors, chronic health conditions, and use of preventive services. Measures of current smoking status, blood pressure screening, height, weight, body mass index, and several other demographic characteristics on the BRFSS have been determined to have high reliability and validity.^33^

The SCD module originated in 2007 with a 10‐question format, evolving by 2015 into a 6‐question module focused on adults aged ≥ 45. This optional module captures state‐level data on the prevalence and impact of SCD, including its interference with daily activities.^34^ The dataset contains prevalence values for each of the 50 states, the District of Columbia, Puerto Rico, the US Virgin Islands, and Guam, which represents an average of point estimates across 2016 to 2021 with states having different total years of data and a median (interquartile range [IQR]) of 2 (1–3) years of measurement per state. We limited this analysis to the 50 states to ensure the availability of the study's exposure variables.

To derive exposure variables, we used the Area Health Resources Files (AHRF) 2020 to 2021 provided by the Health Resources and Services Administration.^35^ AHRF files contain comprehensive data on health resources and services at the state level in the United States. We also derived data from the CDC's state‐level Chronic Disease Indicators database, which relies on BRFSS surveys conducted in 2019 to 2021.^36^ Finally, we used KFF (previously called the Kaiser Family Foundation) State Health Facts data from 2019 to 2022.^37^ KFF is an independent organization committed to providing non‐partisan information^38^ that sources its data from multiple federal databases, such as the US Census American Community Survey.

All data used in this study are deidentified, publicly available without restriction, and do not require institutional review board approval for use.

### 2.2 Outcomes and exposures

The study has two primary outcomes: state‐level prevalence of SCD and SCD‐related functional impairment, both of which come from the “Alzheimer's Disease and Healthy Aging Data.”^31^ SCD is a well‐established construct that has a strong association with future development of dementia.^21^, ^28^, ^30^, ^39^, ^40^, ^41^ Although research suggests that informant‐ or proxy‐reported SCD may be more accurate, the finding is not consistent at a population level.^42^, ^43^, ^44^ We chose SCD‐related functional impairment as the secondary outcome because it may be more specific for objective cognitive impairment than SCD alone.^22^, ^23^, ^24^, ^25^, ^26^, ^27^ The SCD module coded SCD as present when respondents answered affirmatively to the question, “During the past 12 months, have you experienced confusion or memory loss that is happening more often or is getting worse?” The SCD module coded SCD‐related functional impairment as present when respondents with SCD answered “always,” “usually,” or “sometimes” (as opposed to “rarely” or “never”) to the question, “During the past 12 months, how often has confusion or memory loss interfered with your ability to work, volunteer, or engage in social activities outside the home?”

We also report our outcomes within US Census regions (North, West, South, and Midwest) and restricted to certain demographic strata defined by the CDC, including sex (male, female), race/ethnicity (non‐Hispanic White, non‐Hispanic Black, and Hispanic), and age (50–64, ≥ 65).

We selected 12 study exposures to cover a wide range of socioeconomic and medical determinants of cognitive health.^5^, ^6^, ^7^, ^8^, ^9^, ^10^, ^11^, ^12^, ^13^ The study exposures were all state level and included: (1) prevalence of poverty (US Census Bureau definition, 2022), (2) prevalence of unemployment (Bureau of Labor Statistics, March 2022), (3) prevalence of homelessness (US Department of Housing and Urban Development, 2022), (4) median household income (American Community Survey, 2021), (5) prevalence of adults with a college degree (American Community Survey, 2021), (6) prevalence of racial/ethnic minorities (AHRF, 2021‐2022), (7) prevalence of uninsurance (American Community Survey, 2022), (8) physicians per 1000 adults (AHRF, 2016–2020), (9) prevalence of current smoking (CDC Chronic Disease Indicators, 2020), (10) prevalence of hypertension (CDC Chronic Disease Indicators, 2021), (11) prevalence of obesity (CDC Chronic Disease Indicators, 2020), and (12) prevalence of diabetes (CDC Chronic Disease Indicators, 2020). The sources and definitions of each are seen in Table S1 in supporting information. Note that the exposures are not calculated specific to the age group of the outcome variables (≥ 45 years) due to the inability to standardize all exposures to this age group.

**RESEARCH IN CONTEXT**

1. **Systematic review**: The authors reviewed the literature using traditional (e.g., PubMed) sources. Data on outcome variables were extracted from the Centers for Disease Control and Prevention dataset “Alzheimer's Disease and Healthy Aging Data,” which contains state‐level Behavioral Risk Factor Surveillance System (BRFSS) surveys from 2016 to 2021.
2. **Interpretation**: Our findings highlight the critical link between state‐level socioeconomic and medical determinants and prevalence estimates of subjective cognitive decline (SCD) and SCD‐related functional impairment. The strong association between poverty and SCD underscores a crucial, yet often overlooked, aspect of public health efforts in relation to dementia prevention, management, and caregiver support.
3. **Future directions**: This article underscores the importance of integrating medical and social determinants of health within public health strategies addressing cognitive impairment to reduce its burden at a population level. It proposes future research to identify state‐level interventions aimed at reducing dementia as a result of poverty‐directed interventions, as well as supporting impoverished communities bearing the highest burden of living with dementia.

### 2.3 Analytic approach

We created figures of the continental United States with colors representing quintiles of the rates of SCD and SCD‐related functional impairment. We fit individual univariate linear regression models between the 12 exposures and two outcomes. To account for multiple testing, we used a Bonferroni‐corrected threshold for statistical significance of 0.002 (0.05 divided by 24 tests).^45^, ^46^ From the univariate regression models, we derived the Pearson correlation coefficient (rho), coefficient of determination (*R* ^2^), and an associated *p* value. For each exposure–outcome association, we created a scatter plot with a superimposed fitted linear regression line and 95% confidence intervals. We repeated these analyses stratified by sex, race/ethnicity, and age.

We also created a heatmap of the variables showing the correlation coefficients (rho) of their matrices. For the heatmap, we converted all correlations to absolute values such that the strength of association but not the direction is displayed. We further created heatmaps displaying the correlation coefficients (rho) for the outcomes and exposures within the CDC‐defined strata of sex (male, female), race/ethnicity (non‐Hispanic White, non‐Hispanic Black, and Hispanic), and age (45–64, ≥ 65).

We used partial correlation analysis to better understand the influence of each exposure on our outcome measures when all other exposures were held constant. Partial correlation is a statistical technique that measures the degree of association between two variables while controlling for the influence of one or more additional variables. This method is particularly useful in analyses in which multiple interrelated factors are present.^47^, ^48^ As many of the medical determinants of dementia are likely mediators on the causal path between the socioeconomic determinants and outcomes, and vice versa,^49^ the partial correlation coefficients are conservative.

We performed four sensitivity analyses. In the first, we tested a third outcome, SCD‐related dependence, identified by the question “During the past 12 months, as a result of confusion or memory loss, how often have you given up day‐to‐day household activities or chores you used to do, such as cooking, cleaning, taking medications, driving, or paying bills?”^50^ The second sensitivity analysis investigated if the number of years that the SCD module had been administered at the state level affected the findings, which we tested by including a year count variable for the states in the regression model. The third sensitivity analysis uses the prevalence of poverty defined by the supplemental poverty measure instead of the US Census Bureau definition. The supplemental poverty measure extends the official poverty measure by accounting for many government programs that are designed to assist low‐income families, federal and state taxes and work and medical expenses, and geographic variation in poverty thresholds.^51^ The fourth sensitivity analysis reports Spearman rank correlation coefficient in addition to Pearson, to determine whether the findings changed with a non‐parametric versus parametric approach.

All analyses were performed in Stata 18.0 (StataCorp Inc.). We defined significance for the regression models as a two‐tailed *p* < 0.002 (see justification above) and for the partial correlation as *p* < 0.05.

## 3 RESULTS

**FIGURE 1:**
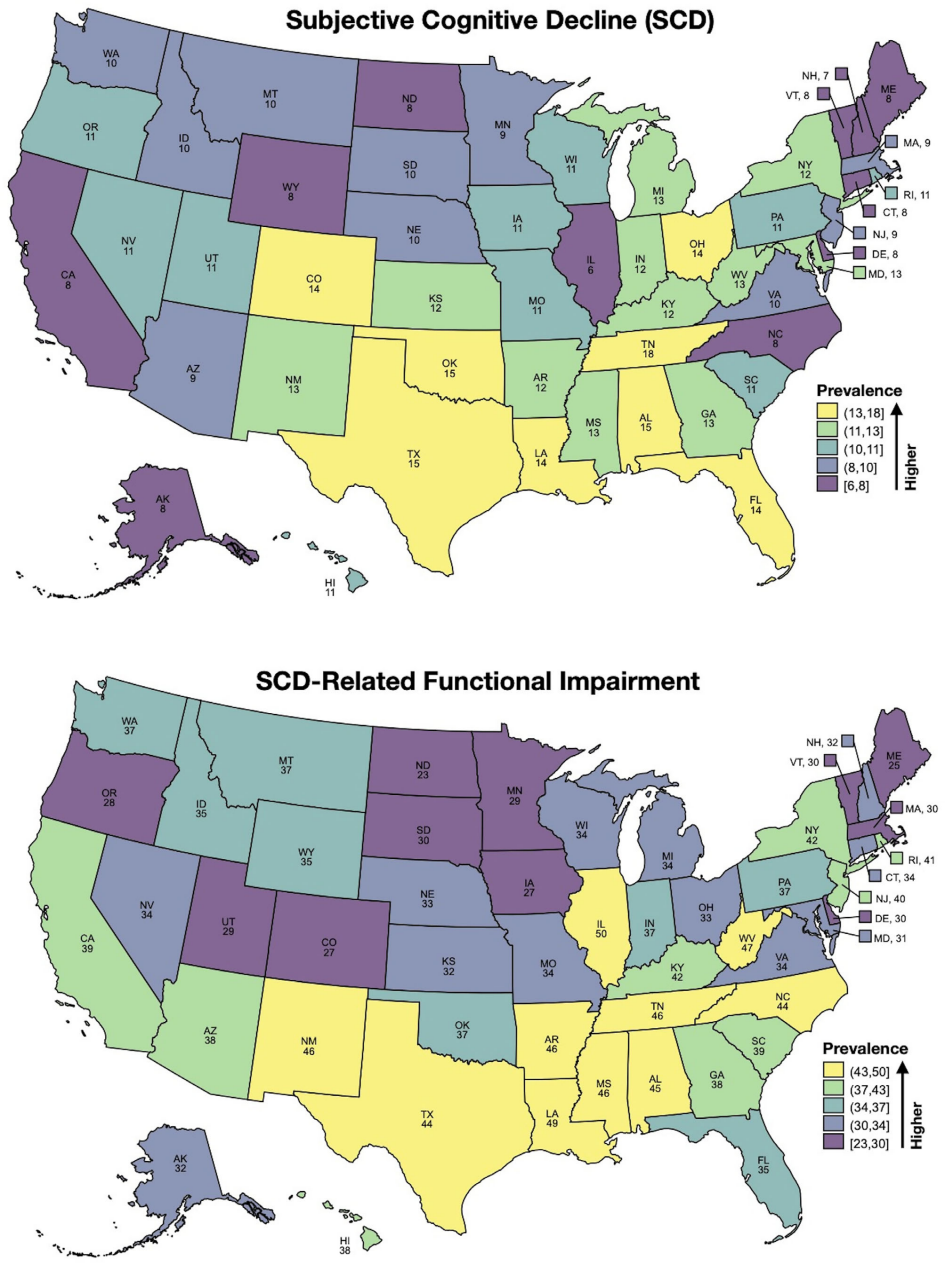
Quintiles by color and prevalence of (top) subjective cognitive decline and (bottom) subjective cognitive decline‐related functional difficulties in the United States. The prevalence as a percentage is shown for each state.

In the 50 states, the median (IQR) prevalence of SCD was 10.8% (9.2–12.8) and, among individuals with SCD, the median (IQR) prevalence of SCD‐related functional impairment was 35.2% (31.9–40.6). The outcomes are shown by quintiles on a map of the continental United States in Figure 1. The raw congruence of SCD and SCD‐related functional impairment deciles between states is 36%, but 70% of states had both outcome measures within one quintile category. The median (IQR) SCD prevalence was highest in the Southern United States at 13.1% (11.5–14.5) versus 9.2% (7.8–10.6) in the North, 10.6% (9.4–12.0) in the Midwest, and 10.3% (9.4–11.3) in the West (*p* = 0.003). Similarly, the median (IQR) prevalence of SCD‐related functional impairment among those with SCD was highest in the Southern United States 43.2% (35.9–46.0) versus 33.6% (30.1–39.8) in the North, 33.1% (29.8–34.2) in the Midwest, and 35.4% (32.0–38.2) in the West (*p* = 0.010).

### 3.1 SCD analysis

**FIGURE 2:**
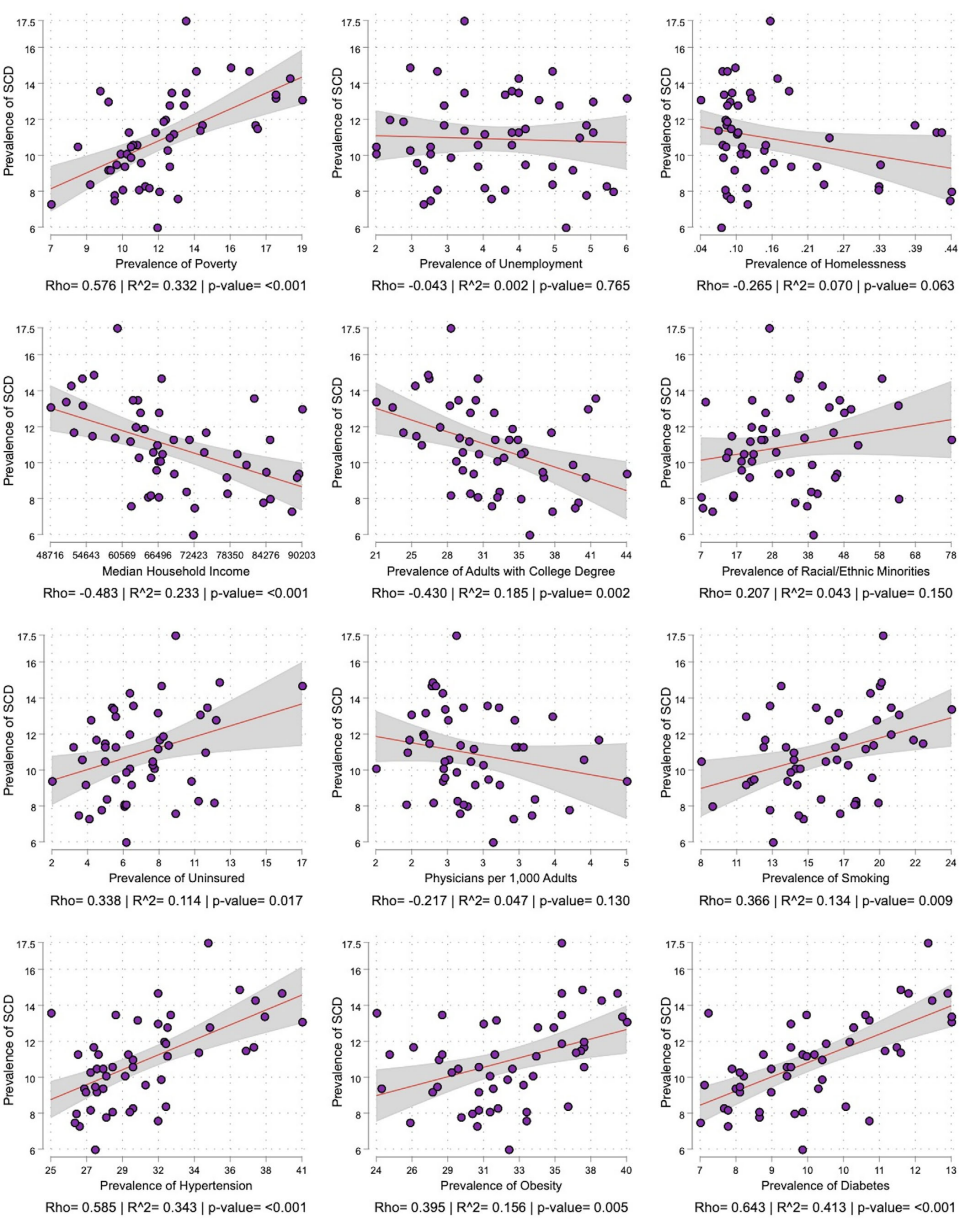
Scatterplot, regression line with 95% confidence interval, correlation coefficient (correlation), and coefficient of determination (*R* ^2^) for the relationship between the prevalence of subjective cognitive decline (SCD) and socioeconomic and medical characteristics of states.

**FIGURE 3:**
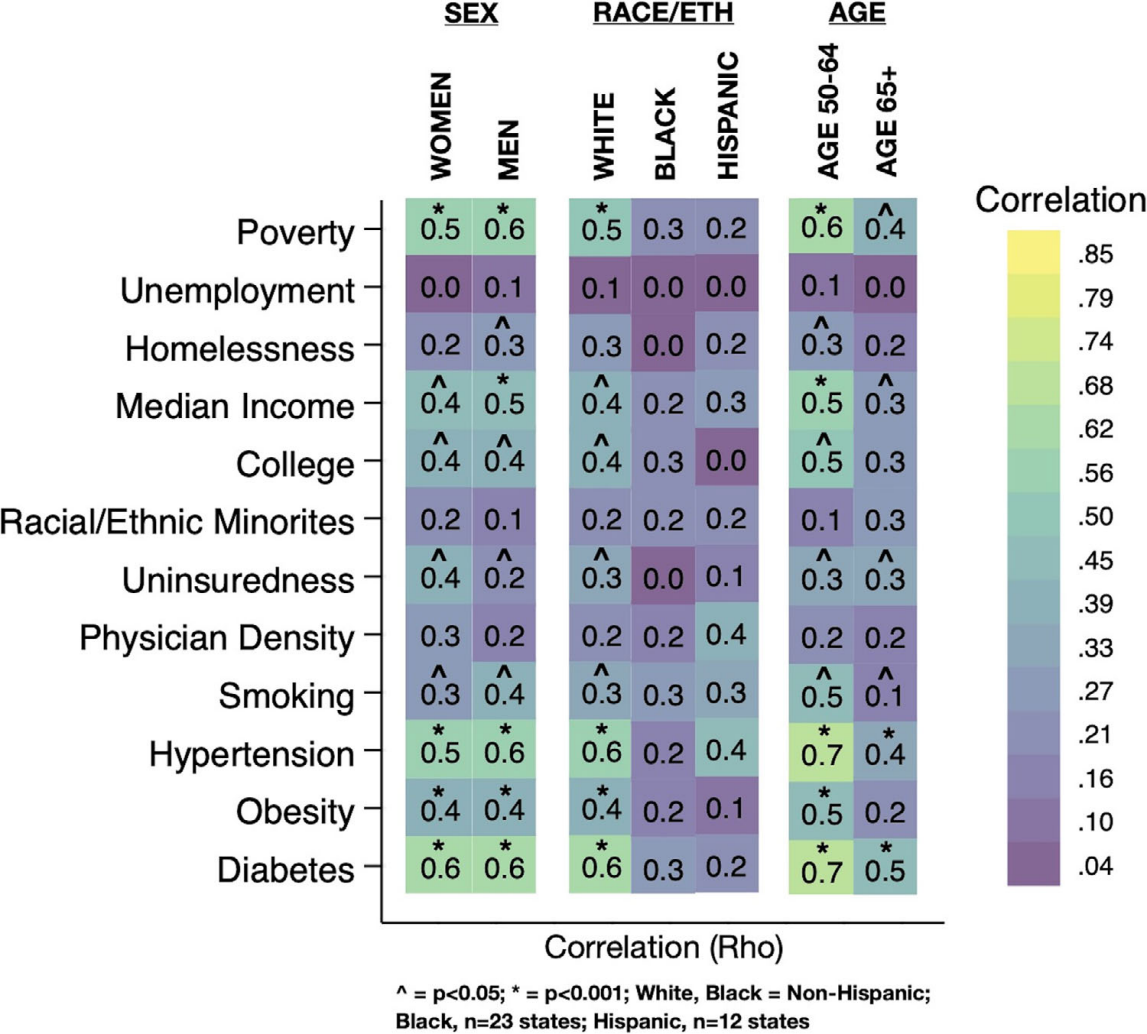
Correlation between subjective cognitive decline and the study exposures after stratifications by sex, race/ethnicity, and age, with all values converted to absolute values such that the strength of association, but not the direction, is displayed.

The univariate correlations between the exposures and SCD are seen in Figure 2. Statistically significant correlations, in order of effect size, included prevalence of diabetes (rho = 0.64, *R* ^2^ = 0.41, *p* < 0.001), hypertension (rho = 0.59, *R* ^2^ = 0.34, *p* < 0.001), poverty (rho = 0.58, *R* ^2^ = 0.33, *p* < 0.001), and median household income (rho = −0.48, *R* ^2^ = 0.23, *p* < 0.001). In the partial correlation analysis, we found that once all variables were held constant the previously associated variables lost significance (Table 1). The heatmap of the univariate correlation of SCD and the exposures within the strata of sex, race/ethnicity, and age are seen in Figure 3. Of note, the strength of the correlations between hypertension, obesity, and diabetes and SCD was higher in non‐Hispanic Whites than Blacks or Hispanics, although the different sample sizes of states between race/ethnicity strata (*n* = 50 for non‐Hispanic White, *n* = 23 for Black, *n* = 12 for Hispanic) may account for this finding. All study exposures had a higher correlation in the age 45 to 64 strata than the age ≥ 65 strata.

**TABLE 1:** Full and partial correlation between the study exposures and subjective cognitive decline.

Variable	Correlation	Partial correlation	*p* value
Prevalence of poverty	0.58	0.06	0.712
Prevalence of unemployment	−0.04	−0.21	0.198
Prevalence of homelessness	−0.27	−0.11	0.491
Median household income	−0.48	−0.001	0.994
Prevalence of adults with college degree	−0.43	−0.08	0.649
Prevalence of racial/ethnic minorities	0.21	0.04	0.803
Prevalence of uninsurance	0.34	0.14	0.399
Physicians per 1000 adults	−0.22	0.16	0.337
Prevalence of current smoking	0.37	0.02	0.900
Prevalence of hypertension	0.59	0.13	0.418
Prevalence of obesity	0.40	−0.27	0.098
Prevalence of diabetes	0.64	0.27	0.091

### 3.2 SCD‐related functional impairment analysis

**FIGURE 4:**
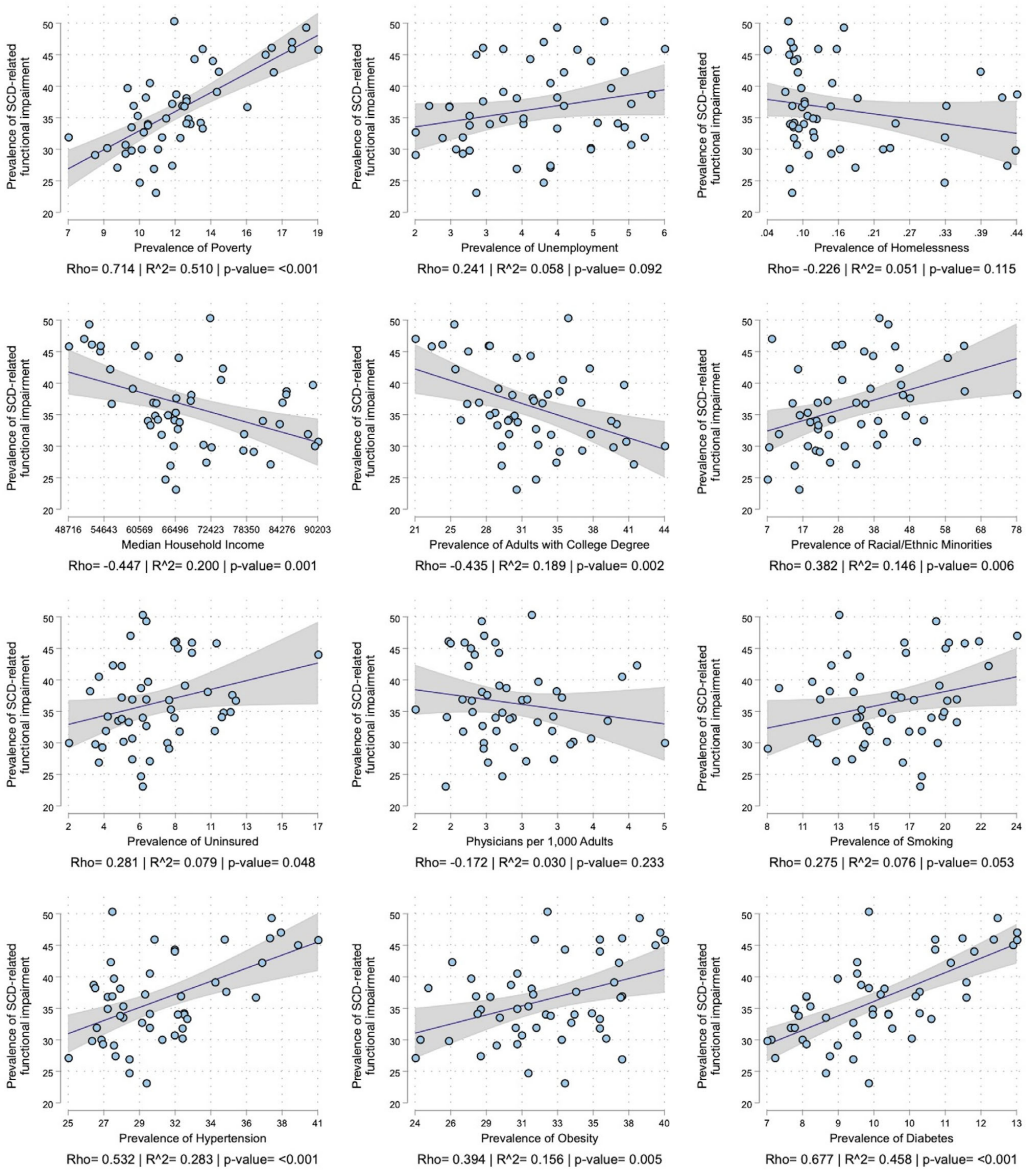
Scatterplot, regression line with 95% confidence interval, correlation coefficient (correlation), and coefficient of determination (*R* ^2^) for the relationship between the prevalence of functional difficulties in individuals with subjective cognitive decline (SCD) and socioeconomic and medical characteristics of states.

**FIGURE 5:**
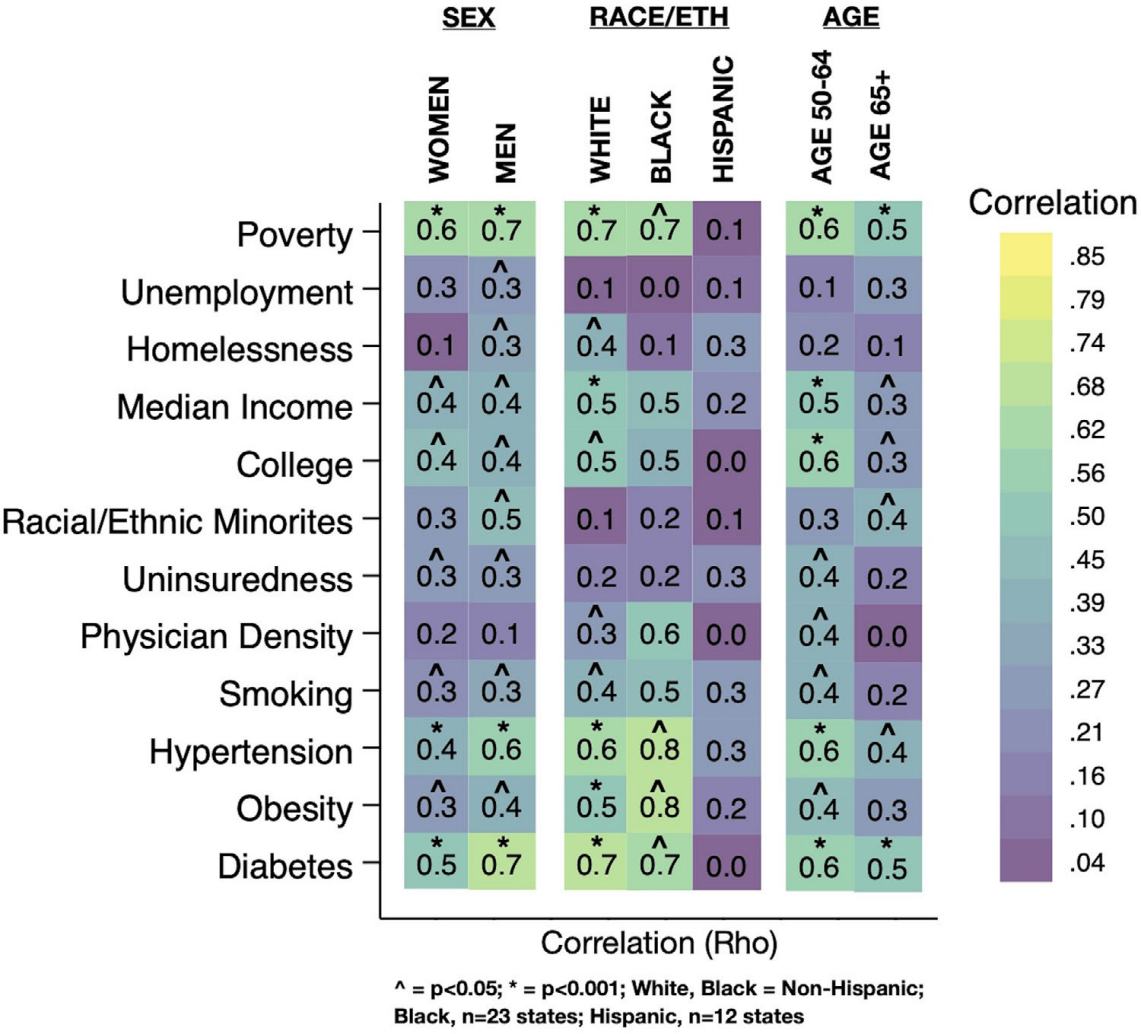
Correlation between subjective cognitive decline‐related functional impairment and the study exposures after stratifications by sex, race/ethnicity, and age, with all values converted to absolute values such that the strength of association, but not the direction, is displayed.

The univariate correlations between the exposures and SCD‐related functional impairment are seen in Figure 4. Statistically significant correlations, in order of effect size, included prevalence of poverty (rho = 0.71, *R* ^2^ = 0.51, *p* < 0.001), diabetes (rho = 0.68, *R* ^2^ = 0.46, *p* < 0.001), hypertension (rho = 0.53, *R* ^2^ = 0.28, *p* < 0.001), and median household income (rho = −0.45, *R* ^2^ = 0.20, *p* = 0.001). However, in the partial correlation analysis we found that once all variables were accounted for, only poverty retained statistical significance (*p* = 0.003; Table 2). The heatmap of the univariate correlations of SCD‐related functional impairment and the exposures within the strata of sex, race/ethnicity, and age are seen in Figure 5. Of note, the strength of the correlations between hypertension, obesity, and diabetes was higher in men, non‐Hispanic Blacks, and all study exposures had a higher correlation in the age 45 to 64 strata than the age ≥ 65 strata.

**TABLE 2:** Full and partial correlation between the study exposures and functional impairment in individuals with subjective cognitive decline.

Variable	Correlation	Partial correlation	*p* value
Prevalence of poverty	0.71	0.47	0.003
Prevalence of unemployment	0.24	−0.10	0.537
Prevalence of homelessness	−0.23	−0.22	0.185
Median household income	−0.45	0.20	0.234
Prevalence of adults with college degree	−0.44	−0.16	0.318
Prevalence of racial/ethnic minorities	0.38	0.03	0.871
Prevalence of uninsurance	0.28	0.08	0.644
Physicians per 1000 adults	−0.17	0.19	0.259
Prevalence of current smoking	0.28	−0.13	0.440
Prevalence of hypertension	0.53	−0.18	0.272
Prevalence of obesity	0.39	0.02	0.902
Prevalence of diabetes	0.68	0.22	0.172

**FIGURE 6 alz14220-fig-0006:**
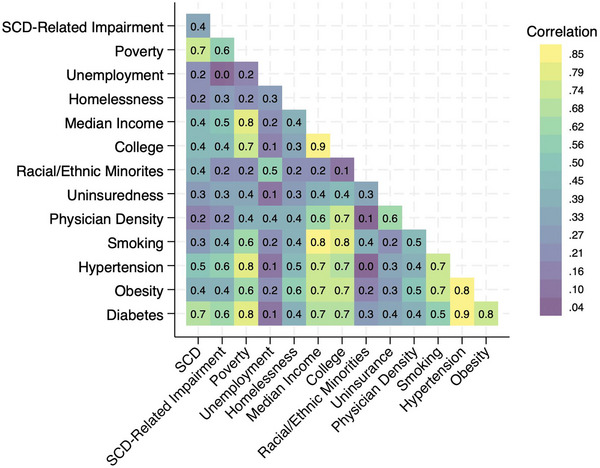
Heatmap of the correlation coefficients between our outcomes and exposures, with all values converted to absolute values such that the strength of association but not the direction is displayed. SCD, subjective cognitive decline.

The heatmap of the full correlation matrix between all the study outcomes and exposures is seen in Figure 6. Among the study outcomes and exposures, we saw the strongest correlation between poverty and SCD‐related functional impairment. The strongest pairwise correlations were seen between median household income and prevalence of adults with college degree (rho = 0.88, *p* < 0.001), hypertension and diabetes (rho = 0.87, *p* < 0.001), and hypertension and obesity (rho = 0.85, *P *< 0.001). The prevalence of poverty was also strongly correlated with diabetes (rho = 0.80, *p* < 0.001).

### 3.3 Sensitivity analyses

In the first sensitivity analysis, the outcome was SCD‐related dependence, which had a median (IQR) prevalence of 31.1% (18.9–48.8). The correlations of our exposures with SCD‐related dependence are shown in Figure S1 in supporting information. Poverty has the strongest association (rho = 0.66, *R* ^2^ = 0.44, *p* < 0.001), followed by diabetes (rho = 0.60, *R* ^2^ = 0.36, *p* < 0.001).

In the second sensitivity analysis, we adjusted our models for the number of years that each state had participated in the SCD module. This did not significantly change the findings reported above (data not shown). In the partial correlation analysis for SCD‐related functional impairment, we continued to find that only poverty retained statistical significance (*p* = 0.004) after adjusting for the number of years that the state had participated in the SCD module.

In the third sensitivity analysis, instead of the official poverty measure we used the supplemental poverty measure, which we also found to be highly associated with state‐level rates of SCD (rho = 0.44, *R* ^2^ = 0.19, *p* = 0.001) and SCD‐related dependence (rho = 0.63, *R* ^2^ = 0.44, *p* < 0.001). In the fourth sensitivity analysis, we found that there were not significant differences in our findings between parametric and non‐parametric tests (Tables S2 and S3 in supporting information).

## 4 DISCUSSION

These results highlight the important[Fig alz14220-fig-0006] role of state‐level prevalence of socioeconomic and medical determinants, principally diabetes, hypertension, and poverty, in quantifying SCD in adults aged ≥ 45 in the United States. Interestingly, while the state‐level prevalence of diabetes and poverty are themselves highly correlated (rho = 0.8), only poverty is independently associated with the outcome of SCD‐related functional impairment after adjusting for all other exposures. This correlation was not found for SCD. While our study was not designed to disentangle the complex causal paths from poverty to our two outcome measures, it is possible that state‐level poverty predisposes individuals to lower functional reserve, which may explain why poverty has a significant partial correlation with SCD‐related functional impairment but not with SCD itself. Although the prevalence of poverty is a complex and confounded exposure, its ability to maintain statistical significance in a model that accounts for all the other study exposures highlights its importance at a geopolitical level.

We also observed regional variation in our outcomes, with the Southern United States showing the highest prevalence of both SCD and SCD‐related functional impairment. These regional disparities are consistent with previous research using the Health and Retirement Study.^52^, ^53^ Our findings reveal a more pronounced association between our study exposures and SCD and SCD‐related functional impairment in individuals aged 45 to 64 years. A potential explanation for this is that mid‐life represents a critical period during which the cumulative effects of earlier lifestyle choices and environmental exposures significantly influence cognitive health, potentially accelerating the onset of cognitive decline if not managed effectively.^6^, ^54^, ^55^, ^56^, ^57^, ^58^, ^59^ It is also possible that mid‐life constitutes a period in which biological mechanisms of resilience—that effectively counteract the detrimental effects of earlier lifestyle choices and environmental exposures—become exhausted.

The traditional focus in cognitive health research is on individual‐level risk factors such as AD pathology and vascular risk factors like hypertension and diabetes.^5^, ^6^, ^7^, ^8^, ^9^, ^10^, ^11^, ^12^, ^13^ The strong association we found between poverty and SCD underscores a crucial, yet often overlooked, aspect of public health efforts in relation to cognitive decline prevention, dementia management, and caregiver support.^60^ Previous research identifies poverty as a likely explanation for the inverse relationship between stroke prevalence and geographic distribution of stroke social services in rural areas.^61^ While individual‐level medical risk factors are clearly important for treatment decisions, at a broader societal, population, and public health level, it is plausible that poverty is the most significant health system and societal risk factor for SCD because it may have downstream effects on all aspects of health and well‐being.^62^, ^63^, ^64^, ^65^, ^66^, ^67^

Even when we accounted for downstream medical determinants, poverty maintained a strong association, suggesting multiple potential underlying mechanisms account for our findings. For instance, the ability to afford items such as blood pressure medications and hearing aids, which can mitigate the risk of dementia, represents just one plausible contributor. This observation aligns with previous epidemiological analyses in related conditions like cardiovascular and cerebrovascular disease, for which socioeconomic factors are consistently associated with the societal burden of disease.^8^, ^68^, ^69^, ^70^, ^71^

Our findings suggest that addressing cognitive impairment requires a multifaceted approach that extends beyond medical solutions to encompass socioeconomic influences. Recognizing poverty as a critical contributor enables the targeting of public health strategies and interventions more effectively, potentially reducing the overall cognitive decline burden at a population level. Such an approach, which integrates socioeconomic and medical determinants, is likely to yield more effective and equitable outcomes.

Previous research shows that social policies in Western countries can have measurable impacts on dementia.^72^ In addition, a critical lesson of the COVID‐19 pandemic relief programs is that US government interventions can immediately lift millions of citizens from poverty.^73^, ^74^ The Finnish Basic Income Experiment also showed that a basic income significantly improves self‐perceived assessments of health and ability to concentrate.^75^ Future research should identify state‐level strategies aimed at reducing dementia as a consequence of poverty‐directed interventions as well as supporting impoverished communities bearing the highest burden of dementia and caring for individuals living with dementia.

### 4.1 Limitations

This study, while extensive, is not without limitations. First, the cross‐sectional design and use of aggregate state‐level data limits our ability to infer causality between the exposures and outcomes due to several factors including inability to ascertain temporality. We cannot infer whether composition or contextual effects underlie the associations found in this study. We also urge caution against interpreting our results at the individual level (the ecological fallacy). There also remains the possibility of reverse causality impacting our effect estimates (i.e., highly prevalent SCD‐related functional impairment causing poverty). The reliance on self‐reported data in the BRFSS as well as regional differences in stigma could introduce response and recall bias, particularly in the assessment of SCD. The variation in the number of years each state participated in the SCD module could also affect the comparability of data across states, though our consistent findings in the sensitivity analysis accounting for years of participation reassures us of the internal validity of our primary analyses’ findings.

Several years of our data also coincide with the COVID‐19 pandemic, which may introduce different biases by state, outcome measure, or exposure measure. As with all observational research, there remains the possibility of residual confounding due to unmeasured or inadequately measured variables. Finally, the associations reported in this analysis are at a state level and are not a proxy for the importance of controlling an individual's risk factors. Further, there may be heterogeneity of factors and outcomes in more granular geographic units within a state that were not accounted for.

## 5 CONCLUSION

This study underscores the imperative of a broader perspective in addressing cognitive impairment in the United States, highlighting the critical importance of socioeconomic factors, particularly poverty. An integrated approach addressing socioeconomic determinants in addition to standard medical risk factors is essential for devising more effective and equitable public health policies and interventions with the potential to reduce the burden of cognitive decline.

## CONFLICT OF INTEREST STATEMENT

Dr. Adam de Havenon received consultant fees from Integra and Novo Nordisk, royalty fees from UpToDate, and has equity in TitinKM and Certus. Dr. Kevin N. Sheth reports compensation from Sense and Zoll, for data and safety monitoring services; compensation from Cerevasc, CSL Behring, Rhaeos, and Astrocyte for consultant services; and a patent for stroke wearables licensed to Alva Health. Dr. Richa Sharma has a provisional patent for methods of training an algorithm to predict ischemic stroke etiology. Dr. Eric L. Stulberg reports no conflicts of interest. Dr. Rebecca F. Gottesman reports no conflicts of interest. Ms. Lauren Littig reports no conflicts of interest. Mr. Ka‐Ho Wong reports no conflicts of interest. Dr. Daniel Sarpong reports no conflicts of interest. Dr. Jeff D. Williamson reports no conflicts of interest. Dr. Nicholas M. Pajewski reports no conflicts of interest. Dr. Adam M. Brickman reports no conflicts of interest. Author disclosures are available in the supporting information.

## CONSENT STATEMENT

All human subjects in the Behavioral Risk Factor Surveillance System provide informed consent to participate in this US government survey.

## Supporting information

Supporting information

Supporting information
