# Supplementary material for: Socioeconomic and medical determinants of state‐level subjective cognitive decline in the United States
Source: Alzheimers Dement. 2024 Oct 1;20(11):7567–79. doi: 10.1002/alz.14220 (PMC11567845; doi:10.1002/alz.14220)
Supplement: Supplementary file 1 — Supporting information [file ALZ-20-7567-s001.docx]

**SUPPLEMENTARY MATERIAL**

**Supplementary Table 1.** Definitions and sources of study exposure data.

| **Exposure** | **Time** | **Source** | **Notes** |
| --- | --- | --- | --- |
| Poverty | 2022 | American Community Survey, 1-Year Estimates | The U.S. Census Bureau’s poverty threshold for a family with two adults and one child was $23,556 in 2022. This is the official poverty measurement used by the Federal Government, and the measure used for most poverty-based data presented on State Health Facts. |
| Supplemental Poverty Measure (SPM) | 2022 | American Community Survey, 1-Year Estimates | The SPM extends the official poverty measure by accounting for many government programs that are designed to assist low-income families but are not included in the official poverty measure. The SPM also includes federal and state taxes and work and medical expenses. In addition, the SPM accounts for geographic variation in poverty thresholds, while the official poverty measure does not. |
| Unemployment | March 2022 | Bureau of Labor Statistics (BLS) | The unemployment rate measures unemployment within the civilian non-institutional population aged 16 years and older. It is seasonally adjusted. Area definitions are based on Office of Management and Budget Bulletin No. 15-01, dated July 15, 2015. |
| Homelessness | 2022 | U.S. Department of Housing and Urban Development, Point in Time Estimates of Homelessness, 2023. | A snapshot of homelessness—both sheltered and unsheltered— on a single night. The one-night counts are conducted during the last 10 days of January each year. Homelessness or homeless refers to the definition set by the US Department of Housing and Urban Development (HUD), which considers an individual aged ≥18 to be homeless if he or she lives in an emergency shelter, transitional housing program (including safe havens), or a place not meant for human habitation, such as a car, abandoned building, or on the streets. |
| Median household income | 2021 | American Community Survey, 1-Year Estimates | In 2021 inflation-adjusted dollars. Data are limited to the household population and exclude the population living in institutions, college dormitories, and other group quarters. For information on confidentiality protection, sampling error, nonsampling error, and definitions of all ACS data, see www.census.gov/programs-surveys/acs/. |
| College degree | 2021 | American Community Survey, 1-Year Estimates | US Census 2021 ACS 5-Year Survey (Table S1501). Adults aged ≥18. |
| Racial/Ethnic Minority | 2016-2020 | Area Health Resources Files, 2021-2022 | Derived from US Census 2020 ACS 5-Year Survey. All ages. |
| Uninsurance | 2022 | American Community Survey, 1-Year Estimates | The ACS asks respondents about their health insurance coverage throughout the previous calendar year. Respondents may report having more than one type of coverage. Data include the civilian noninstitutionalized population in the United States. Adults aged ≥18. |
| Physician density | 2016-2020 | Area Health Resources Files, 2021-2022 | Derived from US Census 2020 ACS 5-Year Survey. |
| Smoking, obesity, and diabetes | 2020 | CDC Chronic Disease Indicators, 2020 | Age-adjusted. Sourced from Behavioral Risk Factor Surveillance System. Smoking - Numerator: Respondents aged ≥18 years who report having smoked ≥ 100 cigarettes in their lifetime and currently smoke every day or some days. Denominator: Respondents aged ≥18 years who reported information about cigarette smoking (excluding unknowns and refusals). Obesity - Numerator: Respondents aged ≥ 18 years who have a body mass index (BMI) ≥30.0 kg/m² calculated from self-reported weight and height. Denominator: Respondents aged ≥ 18 years for whom BMI can be calculated from their self-reported weight and height (excluding unknowns, refusals to provide weight or height, respondents shorter than 3 feet, or 8 feet or taller, respondents weighing less than 50 pounds, or 650 pounds or more, respondents with BMI less than 12 kg/m2, or 100 kg/m2 or greater, and pregnant women). Diabetes - Numerator: Respondents aged ≥18 years ever told by a doctor or other health professional that they have diabetes (excluding women who were told only when pregnant, refusals, and unknowns). Denominator: Respondents aged ≥18 years (excluding unknowns and refusals). |
| Hypertension | 2021 | CDC Chronic Disease Indicators, 2021 | Age adjusted. Sourced from Behavioral Risk Factor Surveillance System. Florida was missing data in 2021, so the value for 2019 was carried forward. Numerator: Respondents aged >18 years who report ever having been told by a doctor, nurse, or other health professional that they have high blood pressure. Women who were told high blood pressure only during pregnancy and those who were told they had borderline hypertension were not included. Denominator: Respondents aged ≥18 years (excluding unknowns and refusals). |

**Supplementary Table 2.** Comparison of parametric (Pearson) and non-parametric (Spearman) correlation coefficients for the outcome of subjective cognitive decline and the study exposures.

| **Variable** | **Parametric Correlation** | **p value** | **Non-parametric**  **Correlation** | **p value** |
| --- | --- | --- | --- | --- |
| Prevalence of poverty | 0.58 | <0.001 | 0.61 | <0.001 |
| Prevalence of unemployment | -0.04 | 0.765 | -0.01 | 0.965 |
| Prevalence of homelessness | -0.27 | 0.063 | -0.23 | 0.115 |
| Median household income | -0.48 | <0.001 | -0.527 | <0.001 |
| Prevalence of adults with college degree | -0.43 | 0.002 | -0.48 | <0.001 |
| Prevalence of racial/ethnic minorities | 0.21 | 0.145 | 0.24 | 0.088 |
| Prevalence of uninsurance | 0.34 | 0.017 | 0.31 | 0.029 |
| Physicians per 1,000 adults | -0.22 | 0.130 | -0.27 | 0.057 |
| Prevalence of current smoking | 0.37 | 0.009 | 0.37 | 0.009 |
| Prevalence of hypertension | 0.59 | <0.001 | 0.60 | <0.001 |
| Prevalence of obesity | 0.40 | 0.005 | 0.43 | 0.002 |
| Prevalence of diabetes | 0.64 | <0.001 | 0.63 | <0.001 |

**Supplementary Table 3.** Comparison of parametric (Pearson) and non-parametric (Spearman) correlation coefficients for the outcome of subjective cognitive decline-related functional impairment and the study exposures.

| **Variable** | **Parametric Correlation** | **p value** | **Non-parametric**  **Correlation** | **p value** |
| --- | --- | --- | --- | --- |
| Prevalence of poverty | 0.71 | <0.001 | 0.69 | <0.001 |
| Prevalence of unemployment | 0.24 | 0.092 | 0.26 | 0.062 |
| Prevalence of homelessness | -0.23 | 0.115 | -0.23 | 0.102 |
| Median household income | -0.45 | 0.001 | -0.43 | 0.002 |
| Prevalence of adults with college degree | -0.44 | 0.002 | -0.43 | 0.002 |
| Prevalence of racial/ethnic minorities | 0.38 | 0.006 | 0.42 | 0.002 |
| Prevalence of uninsurance | 0.28 | 0.048 | 0.30 | 0.038 |
| Physicians per 1,000 adults | -0.17 | 0.233 | -0.22 | 0.126 |
| Prevalence of current smoking | 0.28 | 0.053 | 0.24 | 0.091 |
| Prevalence of hypertension | 0.53 | <0.001 | 0.40 | 0.004 |
| Prevalence of obesity | 0.39 | 0.005 | 0.35 | 0.014 |
| Prevalence of diabetes | 0.68 | <0.001 | 0.65 | <0.001 |

**Supplemental Figure 1.** Scatterplot, regression line with 95% CI, correlation coefficient (correlation), and coefficient of determination (R-squared) for the relationship between the prevalence of needing assistance with day-to-day activities in individuals with self-reported cognitive decline and social and medical characteristics of states.
